# Mobility in Old Age: Capacity Is Not Performance

**DOI:** 10.1155/2016/3261567

**Published:** 2016-02-29

**Authors:** Eleftheria Giannouli, Otmar Bock, Sabato Mellone, Wiebren Zijlstra

**Affiliations:** ^1^Institute of Movement and Sport Gerontology, German Sport University Cologne, Am Sportpark Müngersdorf 6, 50933 Cologne, Germany; ^2^Institute of Physiology and Anatomy, German Sport University Cologne, Am Sportpark Müngersdorf 6, 50933 Cologne, Germany; ^3^Department of Electrical, Electronic, and Information Engineering, University of Bologna, Viale Risorgimento 2, 40136 Bologna, Italy

## Abstract

*Background*. Outcomes of laboratory-based tests for mobility are often used to infer about older adults' performance in real life; however, it is unclear whether such association exists. We hypothesized that mobility capacity, as measured in the laboratory, and mobility performance, as measured in real life, would be poorly linked.* Methods*. The sample consisted of 84 older adults (72.5 ± 5.9 years). Capacity was assessed via the iTUG and standard gait parameters (stride length, stride velocity, and cadence). Performance was assessed in real life over a period of 6.95 ± 1.99 days using smartphone technology to calculate following parameters: active and gait time, number of steps, life-space, mean action-range, and maximum action-range. Correlation analyses and stepwise multiple regression analyses were applied.* Results*. All laboratory measures demonstrated significant associations with the real-life measures (between *r* = .229 and *r* = .461). The multiple regression analyses indicated that the laboratory measures accounted for a significant but very low proportion of variance (between 5% and 21%) in real-life measures.* Conclusion*. In older adults without mobility impairments, capacity-related measures of mobility bear little significance for predicting real-life performance. Hence, other factors play a role in how older people manage their daily-life mobility. This should be considered for diagnosis and treatment of mobility deficits in older people.

## 1. Introduction

With advancing age, it often becomes increasingly difficult to access community resources like grocery stores, doctor's offices, banks, and other essential services and to participate in sociocultural activities. Diminished independent mobility is a predictor of institutionalization [1], falling [2], and dependence and mortality [3] and is inversely associated with quality of life [4, 5] and health status [6]. Independent mobility is therefore a key to successful aging and is routinely assessed by gerontologists and geriatricians.

Mobility is often assessed with established field tests such as the Timed Up-and-Go test [7, 8], the Performance-Oriented Mobility Assessment [9], and the Elderly Mobility Scale [10]. Other common approaches are assessments based upon gait measures [11] and balance tasks [12]. These assessments are reliable since they are performed in a standardized fashion to control for confounding influences; however, they do not necessarily have high construct validity: it remains unclear how well persons' test scores are correlated with their mobility in daily life. Movements of daily life are typically self-initiated, embedded in a rich behavioral context and ecologically valid, while standardized laboratory-type movements are usually initiated by an external “go” signal, are executed in isolation, and serve no ultimate purpose. It has indeed been documented that performance in the laboratory can be substantially different from this in real life [13, 14] and that seniors' performance deficits are sometimes more pronounced in real life than in the laboratory [15] and sometimes less pronounced in real life [13, 16].

Mobility in daily life depends not only on an intact sensorimotor system but also on intact cognition and psychosocial factors. For example, studies have shown that low cognitive status [17], reduced visual attention [9], self-efficacy beliefs [18], and perceived help availability [19] are all associated with reduced mobility in older adults. Again, however, a person's scores on standardized cognitive tests are poorly related to their cognitive performance in real life [20].

The International Classification of Functioning, Disability and Health (ICF), introduced by the World Health Organization (WHO), dissociates between assessments in a standardized-environment, measuring* capacity*, which is indicative of the highest possible level of functioning of an individual in a given domain at a certain moment, and real-life assessments measuring* performance* which is what individuals do in their own current environment [21]. Within the ICF framework, the above work indicates that the known age-related decrease in the* capacity *to be mobile may poorly predict actual mobility* performance*.

With the advent of new miniaturized technology such as GPS and accelerometers, installed in mass-market products such as smartphones and fitness “watches,” objective assessment of everyday in-home as well as out-of-home mobility becomes feasible [22, 23]. Parameters such as number of steps performed, length of active periods, life-space, defined as the area in which an individual moves in a certain time period [24], and other measures have been used to depict the action-range of older adults [25–29]. The present study applies these methods to find out how well real-life mobility is predicted by standard laboratory measures of mobility or, in other words, how closely capacity and performance are linked. We hypothesized that capacity and performance are poorly linked, which would have important implications for the diagnosis and treatment of mobility deficits.

## 2. Methods

Data were collected as part of a cohort study aiming to analyze determinants of daily-life mobility in older adults. All participants underwent a laboratory-based test battery divided into two sessions including several physical, cognitive, social, and psychometric tests as well as an ambulatory mobility assessment. The study has been approved by the Ethics Committee of the German Sport University Cologne, confirming that study design is according to the principles expressed in the Declaration of Helsinki.

### 2.1. Participants

The recruitment strategy included presentations of the project at local senior citizen gatherings, individual invitation letters to persons who expressed interest in participating in studies of the Institute of Movement and Sport Gerontology in the past, and handing out information brochures about the study and individual approach in settings such as local doctor's offices, pharmacies, churches, and senior sport groups. We also contacted assisted-living facilities and if the management showed interest in the project and gave their approval, we presented our project in their facilities and tested persons willing to participate on-site.

In total, 86 persons meeting the criteria for participation in the study were recruited. Inclusion criteria were age older than 65 years, no serious neurological diseases which could interfere with functional mobility, no severe/acute cardiovascular diseases, ability to stand up from a chair independently, a physician's written statement of nonobjection for this person to participate, and an informed consent to the study design.

### 2.2. Standard Laboratory Measures

Mobility capacity was assessed in the laboratory using the extended, instrumented version of the Timed Up-and-Go test [8] (iTUG) [30]. The iTUG is a mobility test, which requires participants to stand up from a chair, walk 7 m at their preferred speed, turn, walk back towards the chair, and sit down again. It was implemented by attaching six inertial measurement units (Opal, APDM Inc., Portland, OR, USA) to the body, two just proximal to the wrists, two just proximal to the ankles, one on the center of the sternum, and one on the waist, approximately above the fifth lumbar vertebra. Each measurement unit contained a triaxial accelerometer, a triaxial gyroscope, and a triaxial magnetometer, whose signals were transmitted via Bluetooth connection to a computer and were processed later by proprietary software package (Mobility Lab*™*), to calculate the parameters: total completion time (iTUG) (s), cadence (steps/min), stride length (m), and stride velocity (m/s). The latter two were determined as the mean of the left and right leg over the 7 + 7 m of straight walking. Each participant completed three trials. The first was considered as practice and the best performance value of the other two trials was used for further analyses.

### 2.3. Real-Life Measures

Mobility performance in real life was assessed using a combination of physical activity and GPS-derived measures via smartphone technology. Participants were given a smartphone (Samsung Galaxy SIII*™*) in an elastic belt and were instructed to don the belt every morning after waking up; they were asked to put the belt around their waist in such a way that the smartphone was located at their back and their body midline. They were requested to leave the smartphone in place until they went to bed at night and to charge it overnight. Data logging was implemented by two applications (apps), one collecting motion sensor data and the other GPS data. Because the majority of participants had not used a smartphone before, they received a manual and about 15 min of familiarization which covered how to turn the smartphone on and off, charge it, use the touch screen, and start the apps. Each participant was offered the opportunity to contact the instructors in case they had questions or faced complications regarding smartphone use. The real-life data recording was conducted between the first laboratory session, where participants received the smartphone, and the second session, where they returned it. We aimed to record the participants' activities for 7 days. However, it was not always possible to organize the sessions exactly 7 days apart. As a result, the total registration time ranged from 6 to 9 days.

Mobility-related activities were recorded via the “uFall” mobile app recently developed within the FARSEEING European research project [31]. The “uFall” app integrates a real-time fall detector which was not enabled in the present study; the app was only used for the continuous recording of the smartphone's raw accelerometer, gyroscope, and magnetometer signals. Recorded data were processed after the registration period and were used to categorize participants' postures and mobility-related activities into different types, such as not worn periods, lying time, sedentary time, active time, and gait time, and to calculate the number of steps. Identification of active and sedentary intervals was performed by means of activity counts and metabolic equivalents (METs) defined in agreement with Sasaki et al. [32]: activity counts were calculated over 1 s time windows. A time window was labelled as “active” when estimated METs were above 1.5 [33]; otherwise, the time window was labelled as “sedentary.” Within active intervals, gait episodes were identified by means of a step detector which is described in the study of Ryu et al. (2013) [34]. Signal processing and features extraction algorithms were implemented in Matlab (The MathWorks Inc., Natick, MA, Release 2012a). Further analyses focused on the following two variables: the sum of active and gait time (AGT) (h) and number of steps (No. of Steps). Since data collection did not target full 24-hour periods and registration times differed between days and participants, we adjusted the data by excluding data which were collected before 7.00 AM and after 9.00 PM as well as data collected on days with activities shorter than 9 hours; AGT and No. of Steps scores were then scaled to fit a 12-hour day and were subsequently averaged across all registration days of a given participant.

**Figure 1:**
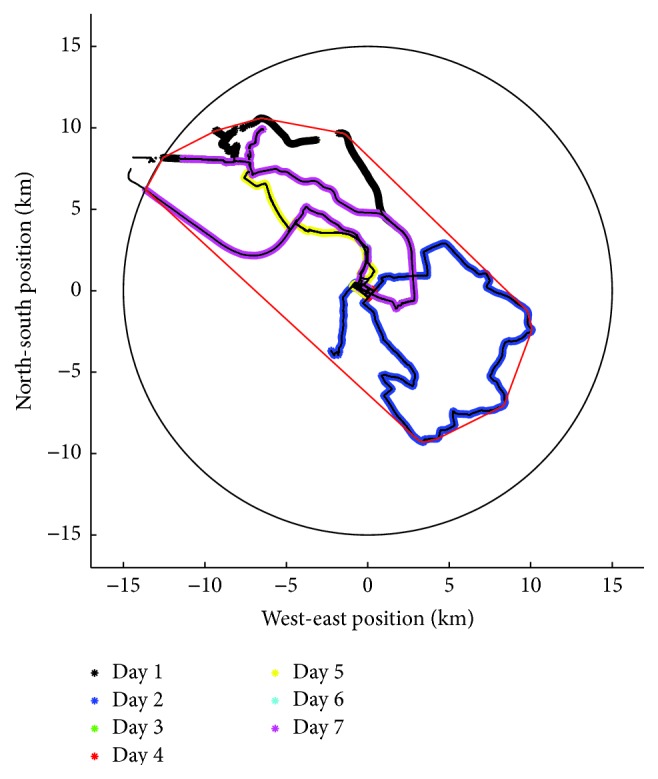
Sample GPS record of one participant demonstrating home location (point (0, 0)) and trajectories for each registration day. The thin red line including all trajectories within the 15 km radius circle represents the parameter “life-space.”

Out-of-home movement was assessed with a self-developed app that collected raw GPS data with a sampling rate of 1 per minute. From these data we calculated the following parameters: life-space (km^2^), the area within which the participants moved during the registration period calculated as the convex hull of all GPS coordinates with Matlab® convhull function; mean action-range (AR-mean) (km), the straight-line distance between the participants' home and the most distal point of each journey, averaged across all journeys during the registration period; and maximum action-range (AR-max) (km), the largest straight-line distance during the registration period. Only data within 15 km around the participants' home (comparable to the size of the greater area of Cologne, Germany, where the study took place) were included in the analysis.  Figure 1 presents a typical example of GPS data obtained over 7 recording days.

### 2.4. Statistical Analyses

The variables “life-space” and “AR-mean” were square-root-transformed to achieve normal distribution. Outliers were identified using the Tukey's outlier filter [35] and removed. Missing data (5.1%) were imputed using the *k*-nearest neighbor algorithm [36]. To make sure that the imputed dataset was not biased, we applied the Little's MCAR test, which showed that data were missing randomly. The hypothesis that laboratory measurements poorly predict daily-life mobility was initially assessed using a correlation approach, looking into the relationships between laboratory and real-life measures. We also conducted a series of stepwise multiple regression analyses in which the five real-life measures (AGT, No. of Steps, life-space, AR-mean, and AR-max) served as dependent variables and the four laboratory measures (iTUG, stride length, stride velocity, and cadence) as predictors. For the stepwise model the limit was 0.10 for entry and 0.05 for removal of variables. For all analyses, the significance level was set at 0.05.

## 3. Results

### 3.1. Mobility Registration Time

The mean number of registration days for the whole sample was 6.95 ± 1.99, with a mean registration time of 70.7 ± 15.00 h for the activity-monitoring data and 104.3 ± 58.5 h for the GPS data.

### 3.2. Descriptive Statistics

From the initial 86 participants two were excluded from the analysis because they did not complete the ambulatory mobility assessment.  Table 1 provides a description of some of the sample's demographics and also summarizes their laboratory as well as real-life measures. Participants were primarily women. Men and women were similar in age. Sixteen percent of the participants were living in assisted-living facilities. Fifty-one percent of the subjects were living alone and only 17% had a higher education degree. Only 3 participants were using gait assistance. In total, 74% of the participants reported health problems (42% were multimorbid and another 32% suffered from a sole disease). The main reported health problems were cardiovascular diseases (42% of the subjects), internal/endocrinological diseases (38%), orthopedic problems (38%), neurological/psychiatric diseases (9%), and others (3%). Regarding use of medication, 59% of the participants reported using medication (34% of the participants used more than one kind of medication and 25% only one kind).

### 3.3. Correlations

Table 2 illustrates that all laboratory measures had significant associations with real-life measures. iTUG, stride length, and stride velocity correlated significantly with at least three of the five real-life measures and also showed the strongest correlations (between *r* = .229 and *r* = .461), while cadence correlated significantly only with AGT. Overall, the correlation coefficients were weak [37].

### 3.4. Multiple Regression Analysis

To evaluate the predictive ability of the laboratory measures for each of the real-life measures five stepwise multiple regression analyses were conducted. Their results are summarized in Tables 3 and 4.

The best model for all real-life measures had only one significant predictor. Overall, the analyses indicated that laboratory measures accounted for a significant but very low [37] proportion of variance (between 5% and 21%) in real-life measures. The best predictors for real-life measures were stride length, which was retained in three models, and iTUG, which was retained in two. Stride velocity and cadence did not contribute significantly to any of the models.

## 4. Discussion

The aim of this study was to examine the predictive ability of standard laboratory measures for real-life mobility and thus also the relationship between capacity and performance measures. The results confirmed our hypothesis that gait measures and mobility tests conducted in the laboratory have very moderate explanatory value for real-life mobility measures and therefore stress the importance of distinguishing between capacity and performance. This evidence highlights the need for real-life mobility-related measures to complement (rather than replace) laboratory measures in geriatric assessments.

As anticipated, the correlation analysis showed significant relationships between the laboratory and the real-life measures. iTUG, stride length, and stride velocity correlated significantly with most of the real-life measures, while cadence correlated significantly only with AGT. Altogether, the measures of real-life mobility-related activities show more and stronger correlations with the laboratory measures than the GPS-derived mobility measures. This can be explained by the fact that the use of assistive devices or other means of transportation like cars, trains, and so forth contribute to the GPS-derived measures, and therefore these measures do not necessarily reflect independent mobility (i.e., walking or bicycling). It is possible that people with lower capacity show larger GPS-derived values due to use of means of transportation other than walking, for example, using car or train rides. If this is the case for some of the participants with low capacity, it would lead to a reduction of the positive correlation between the capacity and the GPS-derived measures of performance. Thus, it is no surprise that laboratory-based capacity measures are more associated with real-life mobility-related activity measures than GPS-derived measures. This is also confirmed by our regression analyses which show that the laboratory measures used in this study explained almost double the variance for AGT and No. of Steps in comparison to life-space, AR-mean, and AR-max. Apparently, factors other than physical capacity play an important role in real-life mobility performance, and especially for life-space related measures of mobility.

While some previous studies found that life-space measures could be predicted by standard measures of functioning, such as gait velocity [28], ADL difficulty [38], and overall physical functioning [29], our comparable measures (stride velocity and iTUG duration) did not. Instead, stride length was the only variable retained in the “life-space” model. This may have to do with the variables included in the regression model. In our study, all models contained partly similar laboratory measures. Gait speed and step length are known to be directly related [39]. Indeed, also in our dataset, stride length correlated significantly with stride velocity (*r* = .653; *p* < .001) as well as with iTUG (*r* = −.538; *p* < .001). Therefore, our results do not contradict the above work.

Among the four capacity measures, iTUG was the strongest and stride length the most consistent predictor for daily-life mobility. The iTUG is a complex task, since it includes demanding mobility-related tasks, which older adults perform in their everyday lives and often have difficulties with (such as standing up and negotiating an object while turning), compared with simple gait variables. Indeed, AGT is the most physically demanding parameter of the real-life mobility parameters measured here, which may explain iTUG being its best predictor. Stride length was the only variable retained to all three life-space models, explaining, however, only a very low (5–11%) proportion of variance. Cadence and stride velocity were not retained in any of the models. This is somewhat surprising, especially for gait speed, as it is considered the most reliable, valid, and specific gait measure [40, 41] and it has been found to be related to physical activity [42–45]. It therefore seems advisable to assess the iTUG, which in addition to its other components includes two walks over 7 meters, rather than only assessing gait during straight walking trajectories, since the combination of iTUG components seems to be more indicative of the requirements for real-life mobility.

Previous research (e.g., [46, 47]) showed somewhat stronger associations between laboratory and real-life measures than our results. However, these studies were primarily based on subjective methods. Results of studies using objective methods in different target groups (e.g., [48–51]) are similar to our findings; only a small percentage of the variance of daily-life mobility is explained by laboratory-based capacity tests. Moreover, a recent study [52] conducted a factor analysis and found that physical capacity measures, similar to the ones used in our study (Sit-to-Stand test, TUG test, and the short Physical Performance Battery), and objective physical activity measures (total duration, number of periods, and mean duration of mobility-related activities) result in two different factors. All of these findings support the hypothesis that standard field tests measuring mobility in laboratory settings and daily-life mobility measures represent different aspects of mobility, each of which has relevance for different domains. Outcomes of capacity tests like the TUG (or the iTUG) inform about fall risk, balance, and functional mobility [8, 53, 54]; on the other hand, real-life physical activity and life-space measures give insight into the extent to which older persons are actively exploiting their capacity. Even when physical capacity is limited, other factors such as the use of assistive devices and/or public transportation may allow older persons to participate in their social context. On the other hand, persons may be inactive, even when their capacity would allow. Obviously other factors than an individual's capacity influence real-life mobility, for example, cognition, mood [17, 55], self-efficacy [18], and weather [56]. Therefore, decisions about interventions aiming to improve mobility in older persons should consider measures of real-life mobility as well as the outcomes of capacity tests. Future studies should further examine the role of different factors on real-life mobility.

Although the current study has the strength of presenting comprehensive mobility patterns of older adults, including long-term real-life physical activity and out-of-home movement measures, we acknowledge several limitations. In order to achieve a performance spectrum as wide as possible we strived to enlist participants living in assisted-living facilities, whose mobility is typically more restricted than this of independent-living older adults. However, only 13 persons (15.5% of the total sample) living in assisted-living facilities could be recruited. Though our sample does present a considerable range of performance at the laboratory measures, it mostly represents community-dwelling older adults living independently without severe mobility impairments. Hence, care should be taken when interpreting our results as they cannot be extended to other populations or adults with severe mobility impairments. Future research should examine the predictive ability of field tests for real-life mobility also in less active samples and/or samples with mobility impairments such as neurological patients or people with cognitive impairments.

Additionally, the real-life data registration period (6.9 ± 1.9 days) varied between participants within the total study period. One of the most important weather parameters which influence physical activity is maximum temperature [48]. In order to control for seasonal variations, we examined the relationship between the average maximum temperature (AMT) for the registration period of each participant and the real-life variables and found that AMT correlates significantly but very weakly (*r* = .184, *p* = .047) only with life-space. However, future studies should aim for a fixed mobility registration period for all the participants to avoid bias [57] due to seasonal variations and in case there are linear relationships between seasonal and mobility variables, seasonal parameters should be controlled for.

## 5. Conclusion

The current study presents mobility patterns in a sample of rather active community-dwelling older adults without severe mobility impairments based on combination of capacity and performance measures and shows that standard laboratory-based tests have limited predictive ability for real-life mobility. This shows that capacity and performance represent different aspects of mobility. Therefore, comprehensive mobility assessments should include capacity measures as well as measures of real-life out-of-home mobility. Additionally, as anticipated, this study confirms that physical activity is better explained by physical functioning, when compared with life-space measures. Finally, this study highlights the utility of the iTUG and, considering its rather simple execution, it suggests that it should be preferred over simple gait measures, as it explains more aspects and higher proportion of real-life mobility.

## Figures and Tables

**Table 1:** Participants' descriptive data and laboratory- and real-life measures.

	Mean	SD	Min	Max
Age (total sample)	72.5	5.9	65	88
Men (*n* = 31)	72.4	5.8	65	88
Women (*n* = 53)	72.5	5.9	65	88
BMI	24.1	3.2	16.7	33.1

iTUG (s)	16.0	2.6	10.5	27.5
Stride length (m)	1.38	0.13	1.01	1.65
Stride velocity (m/s)	1.34	0.16	0.82	1.76
Cadence (steps/min)	115.9	10.4	87.8	145.2

AGT (h)	4.3	0.9	1.8	6.2
No. of Steps	11042	3474	3903	20890
Life-space (km^2^)	52.9	43.8	0.2	178.2
AR-mean (km)	1.4	1.0	0.1	3.8
AR-max (km)	10.4	4.2	0.5	15.0

Mean: average values; SD: standard deviation; Min: minimum values; Max: maximum values; BMI: body mass index; iTUG: instrumented Timed Up-and-Go test; AGT: active and gait time; No. of Steps: number of steps; AR-mean: average action-range; AR-max: maximum action-range.

**Table 2:** Associations between laboratory and real-life measures (Pearson's correlation coefficients, *r* (^*∗*^ *p* < .05; ^*∗∗*^ *p* < .01)).

	AGT	No. of Steps	Life-space	AR-mean	AR-max
iTUG	−.461^*∗∗*^	−.442^*∗∗*^	−.295^*∗∗*^	−.199	−.229^*∗*^
Stride length	.266^*∗*^	.369^*∗∗*^	.331^*∗∗*^	.232^*∗*^	.234^*∗*^
Stride velocity	.396^*∗∗*^	.421^*∗∗*^	.273^*∗*^	.213	.130
Cadence	.261^*∗*^	.185	.034	.052	−.036

AGT: active and gait time; No. of Steps: number of steps; AR-mean: average action-range; AR-max: maximum action-range; iTUG: instrumented Timed Up-and-Go test.

**Table 3:** Significant predictors and their standardized regression coefficients for the mobility-related activity measures.

AGT		No. of Steps	
Predictors	Beta	Predictors	Beta
iTUG	−.461^*∗∗∗*^	iTUG	.442^*∗∗∗*^
*F*(1,82) = 22,155		*F*(1,82) = 19,894	
*R* ^2^ = .213^*∗∗∗*^		*R* ^2^ = .195^*∗∗∗*^	

Bottom row: degrees of freedom and coefficients of determination (*R* ^2^) for each model.

^*∗∗∗*^ *p* < .001.

AGT: active and gait time; No. of Steps: number of steps; iTUG: instrumented Timed Up-and-Go test.

**Table 4:** Significant predictors and their standardized regression coefficients for the GPS-derived measures.

Life-space		AR-mean		AR-max	
Predictors	Beta	Predictors	Beta	Predictors	Beta
Stride length	.331^*∗∗*^	Stride length	.231^*∗*^	Stride length	.233^*∗*^
*F*(1,82) = 10,094		*F*(1,82) = 4,625		*F*(1,82) = 4,713	
*R* ^2^ = .110^*∗∗*^		*R* ^2^ = .053^*∗*^		*R* ^2^ = .054^*∗*^	

Bottom row: degrees of freedom and coefficients of determination (*R* ^2^) for each model.

^*∗*^ *p* < .05; ^*∗∗*^ *p* < .01.

AR-mean: average action-range; AR-max: maximum action-range.
